# Altered Melatonin Secretion and Recurrent Sleep-Related Headache in a Patient With a Pineal Cyst: A Case Report

**DOI:** 10.7759/cureus.117474

**Published:** 2026-10-05

**Authors:** Oneda Çibuku, Kjanda Elpenoria, Vasilika Thimjo, Elda Caushllari, Ergys Xhukellari

**Affiliations:** 1 Neurovascular Department, University Hospital Center "Mother Teresa" (QSUT), Tirana, ALB; 2 Neurology, Fier Regional Memorial Hospital, Fier, ALB; 3 Neurology, University of Medicine, Tirana, Tirana, ALB

**Keywords:** circadian rhythm, melatonin, nocturnal headache, pineal cyst, pineal gland dysfunction, sleep-related headache

## Abstract

Melatonin contributes to the circadian regulation of sleep and nociception, and disruption of its secretion has been implicated in headache disorders with nocturnal periodicity. The functional relevance of pineal cysts in this context remains poorly defined. A 23-year-old woman presented with a four-year history of stereotyped headaches occurring exclusively between 1:30 and 4:00 a.m. and awakening her from sleep. The attacks were occipital-suboccipital, pulsating and pressure-like, rated 7/10 in intensity, and accompanied by scalp allodynia, bilateral conjunctival hyperemia and lacrimation, mild nasal congestion, and restlessness. Neurologic examination and investigations for secondary causes were unremarkable. Serial brain magnetic resonance imaging (MRI) demonstrated a benign-appearing pineal cyst measuring initially 21 × 11 × 10 mm, without hydrocephalus, obstruction of cerebrospinal fluid pathways, or aggressive radiologic features. Two melatonin measurements obtained at 3:00 a.m. were reduced at 14 and 16 pg/mL against a laboratory reference range of 40-80 pg/mL. Treatment with oral melatonin at 4 mg nightly reduced the headache symptoms by approximately 50% within four weeks. Increasing the dose to 8 mg nightly was followed by complete resolution within six weeks. After three months, the nocturnal melatonin concentration was 52 pg/mL, and the patient remained headache-free without reported adverse effects at the six-month follow-up. This case demonstrates a temporal association among nocturnal headaches, reduced nocturnal melatonin secretion, and improvement following melatonin supplementation. Although a single case cannot establish causality, assessment of circadian hormone secretion may be considered in selected patients with pineal lesions and strictly nocturnal headaches.

## Introduction

Headache disorders with a strictly nocturnal pattern highlight the interaction between circadian biology and pain modulation. Melatonin, primarily secreted by the pineal gland during the biological night, contributes to circadian timing and sleep regulation and has been implicated in pain modulation [1-3]. Alterations in melatonin secretion have been reported in primary headache disorders, including migraine and trigeminal autonomic cephalalgias [1,3,4].

Pineal cysts are frequently detected as incidental findings on brain MRI and are generally considered benign and asymptomatic. An association between pineal cysts and headaches has been reported, although causality remains uncertain [5]. Melatonin secretion has also been investigated in patients with pineal cysts, and a previous case report described nocturnal headaches, a low nocturnal melatonin concentration, and improvement following supplementation [6].

We present a patient with strictly nocturnal headaches, a benign-appearing pineal cyst, and low melatonin concentrations on two measurements obtained at 3:00 a.m., whose symptoms resolved following melatonin supplementation.

## Case presentation

A 23-year-old woman with no previous neurologic history presented with a four-year history of strictly nocturnal headaches. The attacks occurred abruptly between 1:30 and 4:00 a.m. and awakened her from sleep. The pain was occipital-suboccipital, pulsating and pressure-like, and rated 7/10 in intensity. Associated symptoms included scalp allodynia, bilateral conjunctival hyperemia and lacrimation, mild nasal congestion, and restlessness. She denied photophobia, phonophobia, nausea, and daytime headaches. The neurologic examination was normal.

Baseline brain magnetic resonance imaging (MRI) demonstrated a cystic lesion in the pineal region measuring 21 × 11 × 10 mm, with minimal peripheral wall enhancement on postcontrast images. There was no evidence of hydrocephalus or obstruction of the cerebrospinal fluid (CSF) pathways.

MRI performed one year later showed that the lesion was stable in size and morphology, measuring 21 × 11 × 10 mm. Minimal mural enhancement remained visible on postcontrast sequences. Representative MRI findings are shown in Figure 1.

**Figure 1:**
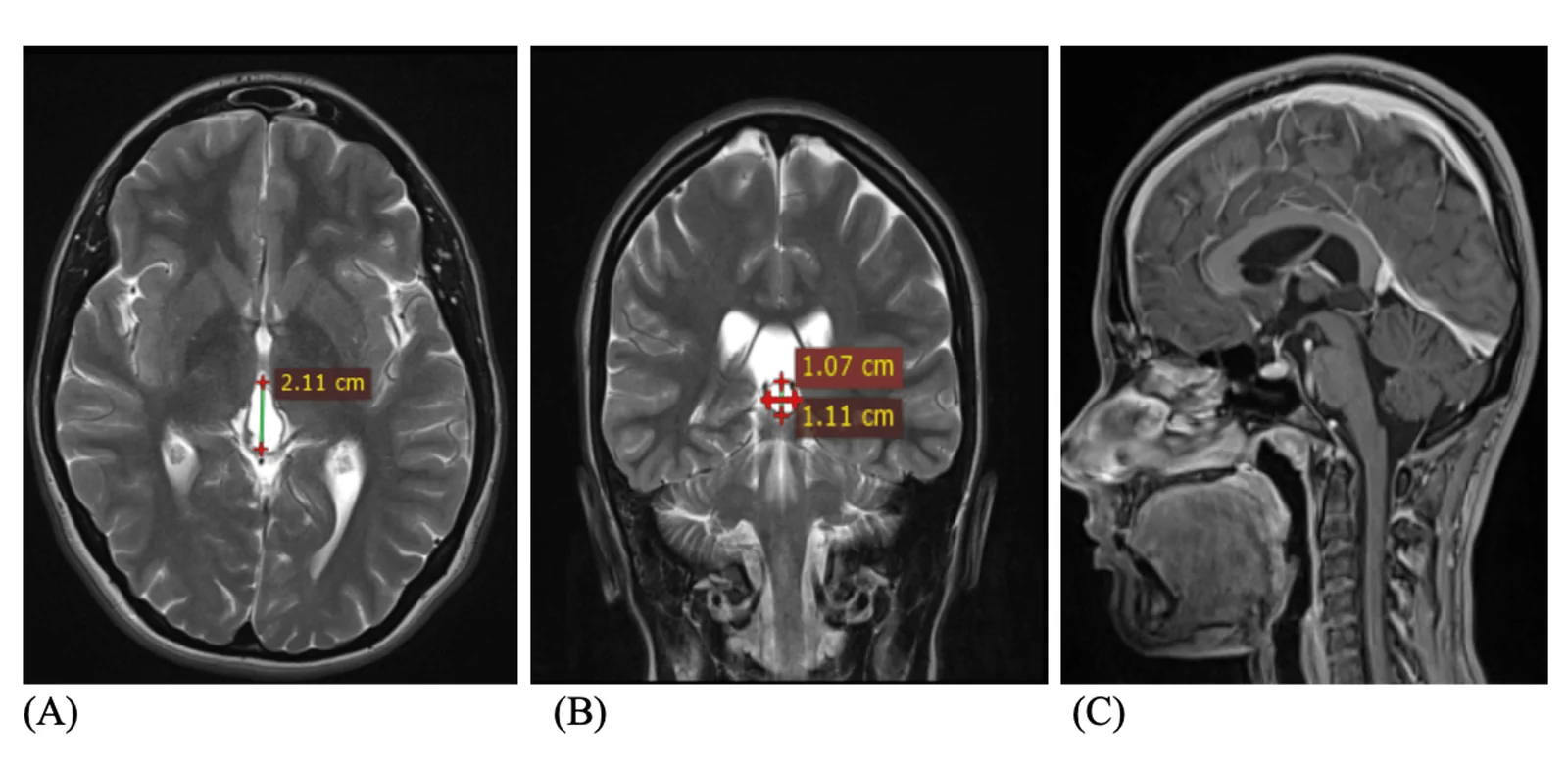
Magnetic resonance imaging of the brain demonstrating a pineal region cyst. T2-weighted axial (A) and coronal (B) images show a hyperintense cystic lesion measuring 21 × 11 × 10 mm. A postcontrast T1-weighted sagittal image (C) demonstrates minimal mural enhancement.

A third MRI examination demonstrated a slight interval increase in lesion size to 23 × 12 × 10 mm, representing an increase of approximately 2 mm in the craniocaudal dimension. No wall enhancement was observed on post-contrast sequences, in contrast to the minimal peripheral enhancement seen on the first two examinations. There was no mural nodularity or other radiologic evidence of aggressive behavior.

A comprehensive laboratory evaluation was performed to investigate inflammatory, autoimmune, endocrine, neoplastic, and infectious causes of the patient's symptoms. The available records did not identify a secondary systemic cause.

Because of the strictly nocturnal headache pattern and the presence of a pineal region lesion, nocturnal melatonin testing was performed. Two pretreatment measurements obtained at 3:00 a.m. showed reduced melatonin concentrations of 14 and 16 pg/mL relative to the laboratory reference interval of 40-80 pg/mL. The results are summarized in Table 1.

**Table 1:** Nocturnal melatonin measurements before and after supplementation

Assessment	Melatonin concentration	Unit	Sampling information	Laboratory reference range	Interpretation
First pretreatment measurement	14	pg/mL	3:00 a.m.	40–80 pg/mL	Decreased
Repeat pretreatment measurement	16	pg/mL	3:00 a.m.	40–80 pg/mL	Decreased
Three-month follow-up	52	pg/mL	Nocturnal; exact time not recorded	40–80 pg/mL	Within reference range

Treatment with oral melatonin at 4 mg nightly reduced headache symptoms by approximately 50% within four weeks. The dose was subsequently increased to 8 mg nightly, resulting in complete resolution within six weeks. At three months, the nocturnal melatonin concentration was 52 pg/mL. The pretreatment and follow-up measurements are shown in Figure 2. At the six-month follow-up, the patient remained headache-free and reported no adverse effects. 

**Figure 2:**
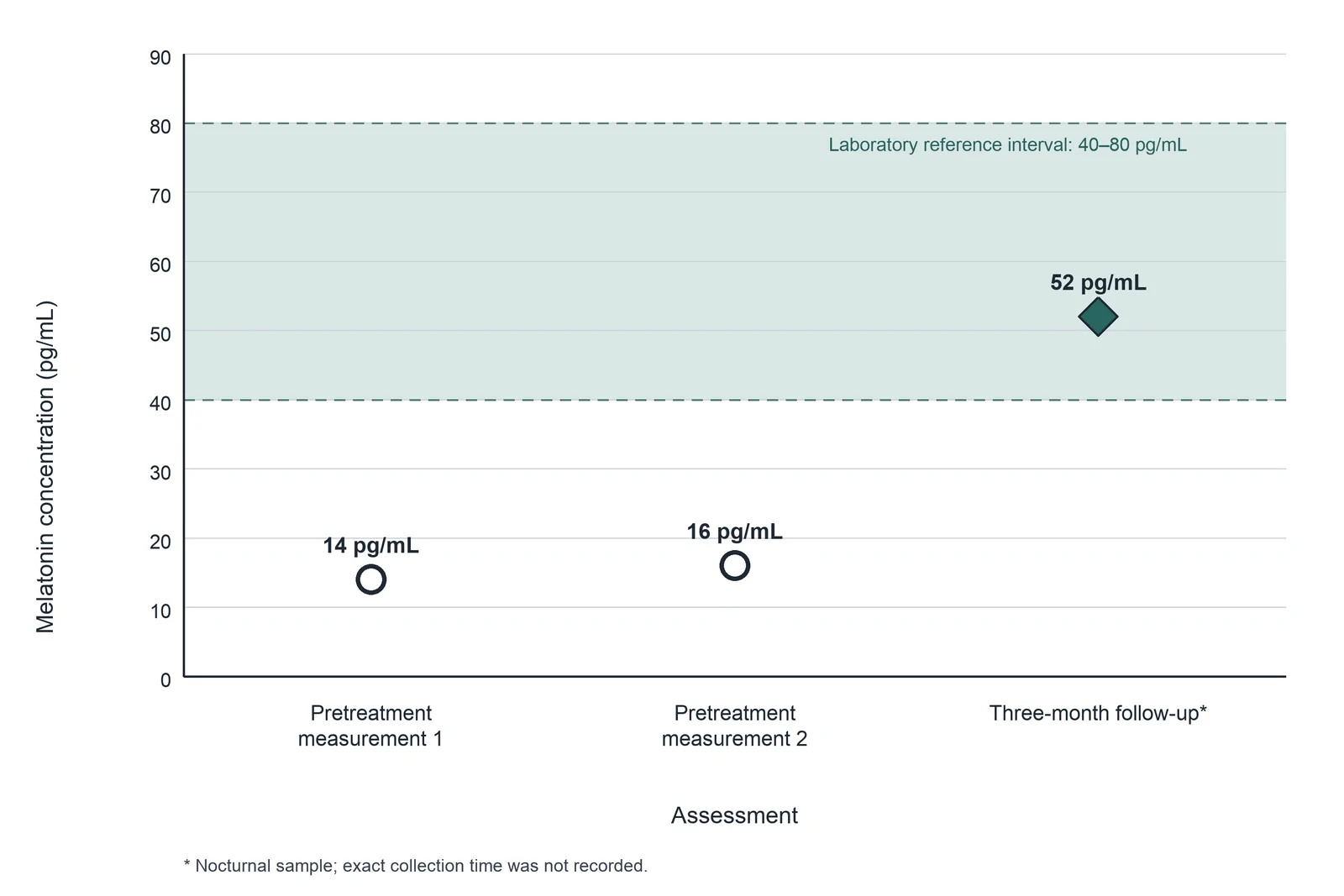
Nocturnal melatonin concentrations before and after melatonin supplementation. The two pretreatment concentrations measured at 3:00 a.m. were 14 and 16 pg/mL. The nocturnal concentration measured after three months of supplementation was 52 pg/mL. The shaded area represents the laboratory reference interval of 40–80 pg/mL. The exact collection time of the follow-up sample was not recorded.

## Discussion

Although pineal cysts have traditionally been regarded as incidental findings, emerging evidence challenges the notion that they are universally asymptomatic in the absence of hydrocephalus or Parinaud’s syndrome [5,6]. Earlier neurosurgical classifications defined symptomatic pineal cysts only in the context of three clinical syndromes: paroxysmal headache with gaze paresis, chronic headache with papilledema and hydrocephalus, or pineal apoplexy. However, several recent series and case-control studies indicate that even non-hydrocephalic patients may experience headache patterns that correlate with the presence of a pineal cyst, independent of its size. In one study of 51 non-hydrocephalic patients, headache frequency was nearly double compared with age- and sex-matched controls, suggesting a functional rather than mass-effect mechanism [5].

The differential diagnosis of headaches arising during sleep includes hypnic headache, trigeminal autonomic cephalalgias, and migraine occurring during sleep. Under ICHD-3, definite hypnic headache requires attacks arising exclusively during sleep and causing awakening, occurring on at least 10 days per month for more than three months, lasting 15 minutes to four hours after waking, and occurring without cranial autonomic symptoms or restlessness. The autonomic manifestations and restlessness reported in this patient therefore argue against definite hypnic headache. Cluster headache, paroxysmal hemicrania, and SUNCT/SUNA warrant consideration because of the autonomic features; however, the occipital-suboccipital location and bilateral ocular manifestations are atypical. These disorders differ in attack duration and frequency, and paroxysmal hemicrania additionally requires an absolute response to indomethacin. The absence of nausea, photophobia, and phonophobia argues against definite migraine without aura. Incomplete documentation of attack duration, frequency, and pain laterality limits formal classification; consequently, “sleep-related headache” is used descriptively. Secondary causes, including sleep apnea, nocturnal hypertension, hypoglycemia, medication overuse, and intracranial disease, also require consideration. Response to melatonin is not diagnostic and does not establish a causal relationship between the cyst and the headaches. [7]

Multiple hypotheses have been proposed to explain this association. Among them, altered melatonin secretion has gained particular traction [1-3,8]. The pineal gland is a critical node in the hypothalamic-pineal axis, and even small structural alterations-short of producing mass effect-may disrupt the normal circadian rhythm of melatonin release. Dysregulated melatonin secretion is known to impair nocturnal antinociception, lower the threshold for trigemino-vascular activation, and facilitate the emergence of strictly nocturnal or early-morning headache phenotypes [3,9]. 

Other proposed mechanisms, such as intermittent aqueductal obstruction or altered intracranial pressure pulsatility, have been described in small physiological studies, with some reports showing increased pulsatile ICP and MRI biomarkers suggestive of central venous hypertension. Nevertheless, these findings remain inconsistent, and their clinical relevance is uncertain outside of neurosurgical cohorts [6]. Importantly, the majority of studies supporting these mechanisms derive from small, heterogeneous samples with limited quantitative headache characterization and without adequate controls.

The neurosurgical literature often debates the role of surgical decompression; however, indications remain controversial and largely restricted to patients with hydrocephalus, progressive cyst enlargement, or Parinaud’s syndrome. Only a minority of neurosurgeons consider operating for isolated headache, reflecting the absence of standardized criteria, the lack of controlled studies, and high variability in reported outcomes. Moreover, symptom improvement following surgery in non-specific headache cases does not necessarily prove causality, especially given the retrospective design and potential for reporting bias.

This case describes the coexistence of a pineal cyst, low melatonin concentrations at two nocturnal sampling points, and headache remission following supplementation. These observations are hypothesis-generating and do not establish that the cyst impaired melatonin secretion or caused the headaches. Improvement with melatonin may reflect therapeutic effects independent of the cyst. The patient exhibited 1) precise nocturnal timing of attacks, 2) biochemical evidence of reduced nocturnal melatonin levels [1-4], and 3) complete symptom remission following melatonin supplementation [3,10].

Two pretreatment measurements obtained at 3:00 a.m. cannot determine the phase, amplitude, duration, or peak of the melatonin rhythm. Low concentrations at this time could reflect reduced secretion or an advanced or delayed circadian phase. The undocumented follow-up collection time prevents direct comparison with baseline, and measurement during supplementation cannot demonstrate restoration of endogenous secretion. Serial sampling under standardized conditions, including salivary melatonin profiling or timed urinary 6-sulfatoxymelatonin measurements, would provide a more informative assessment.

## Conclusions

This case suggests a hypothesis-generating association among a pineal region cyst, low melatonin concentrations at two nocturnal sampling points, and sleep-related headache. The stereotyped nocturnal phenotype, reduced pretreatment melatonin concentrations, and sustained remission after supplementation provide a biologically plausible basis for functional pineal involvement in the absence of hydrocephalus or overt mass effect.

A temporal association in a single patient cannot establish causality or exclude nonspecific treatment effects. Prospective controlled studies incorporating standardized headache phenotyping, serial neuroimaging, circadian hormone profiling, and objective sleep assessments are needed to clarify the underlying pathophysiology and determine when melatonin supplementation may be appropriate.
